# No Evidence of Narrowly Defined Cognitive Penetrability in Unambiguous Vision

**DOI:** 10.3389/fpsyg.2017.00852

**Published:** 2017-07-10

**Authors:** Nikki A. Lammers, Edward H. de Haan, Yair Pinto

**Affiliations:** ^1^Department of Brain and Cognition, University of AmsterdamAmsterdam, Netherlands; ^2^Department of Neurology, Academic Medical CentreAmsterdam, Netherlands

**Keywords:** high-level factors, bottom-up progressing, visual illusion, peripheral vision, cognitive penetrability, uniformity illusion, modular system, perception

## Abstract

The classical notion of cognitive impenetrability suggests that perceptual processing is an automatic modular system and not under conscious control. Near consensus is now emerging that this classical notion is untenable. However, as recently pointed out by Firestone and Scholl, this consensus is built on quicksand. In most studies claiming perception is cognitively penetrable, it remains unclear which actual process has been affected (perception, memory, imagery, input selection or judgment). In fact, the only available “proofs” for cognitive penetrability are proxies for perception, such as behavioral responses and neural correlates. We suggest that one can interpret cognitive penetrability in two different ways, a broad sense and a narrow sense. In the broad sense, attention and memory are not considered as “just” pre- and post-perceptual systems but as part of the mechanisms by which top-down processes influence the actual percept. Although many studies have proven top-down influences in this broader sense, it is still debatable whether cognitive penetrability remains tenable in a narrow sense. The narrow sense states that cognitive penetrability only occurs when top-down factors are flexible and cause a clear illusion from a first person perspective. So far, there is no strong evidence from a first person perspective that visual illusions can indeed be driven by high-level flexible factors. One cannot be cognitively trained to see and unsee visual illusions. We argue that this lack of convincing proof for cognitive penetrability in the narrow sense can be explained by the fact that most research focuses on foveal vision only. This type of perception may be too unambiguous for transient high-level factors to control perception. Therefore, illusions in more ambiguous perception, such as peripheral vision, can offer a unique insight into the matter. They produce a clear subjective percept based on unclear, degraded visual input: the optimal basis to study narrowly defined cognitive penetrability.

## Believing is Seeing

Why do we see things the way we do? This fundamental question of how perceptual input is translated into a subjective experience of the world has been discussed for decades. We effortlessly perceive a rich visual world, even though sensory input is often noisy or unreliable. For example, in peripheral vision large numbers of rods provide input to only a single ganglion cell. Therefore, in peripheral vision retinal input is fairly crude and less sensitive to color information than the fovea (Westheimer, 1982; Anderson et al., 1991). Yet we perceive the world as rich in color and detail (Lamme, 2006; Block, 2007, 2011; Rahnev et al., 2011). So how can human perceptual experience be so clear, when it is often based on unclear input?

There are currently two major, but conflicting, answers on the question why we see things the way we do. The first answer is the classical bottom-up view. The classical view states that our visual experience is purely based on a sensory/bottom-up signal, translated according to fixed rules (that may involve world knowledge). A highly influential psychologist in this regard is J. J. Gibson (1904–1979). Gibson states that vision is purely based on information from the environment and that it is not affected by cognitive construction or processing. Gibson’s view is also known as ecological psychology (Gibson, 1966). This bottom-up processing is often considered as *cognitively impenetrable*. Cognitive impenetrability can be defined as the inability to consciously and purposefully modulate the processing of a mental operation that is thought to be carried out in an automated unsupervised manner, such as basic sensory perception. This modular system is domain specific and its operation is mandatory (Fodor, 1983). Although some theories about the visual system are based on this concept, the classical view cannot clearly explain how noisy input is often experienced as a rich visual percept and how object recognition is influenced by contextual information [see, e.g., Bar (2004) for a review on object perception]. It seems that theories based on purely bottom-up processing (without any influence of top-down processes) do not hold, and have become outdated.

The second answer to the question why we see things the way we do is the alternative top-down view. In contrast to the classical view, the alternative view states that our perception is affected by transient internal states, such as wishes, expectations and beliefs. This latter view, also known as cognitive penetrability (CP), claims that (intentions of) actions can change our perception through flexible priors. Higher-level cognitive states routinely penetrate our perception, such that what we see is an alloy of bottom-up factors and beliefs, desires and motivations. The brain continually updates its model of the world based on a Bayesian weighing of sensory input (bottom-up) and prior expectations (top-down) (Knill and Pouget, 2004; Clark, 2013; Summerfield and de Lange, 2014; Pinto et al., 2015). Our perception is cognitively modulated in many ways, for instance, in brightness illusions (Adelson, 1993), Ramachandran’s scotoma (Ramachandran and Gregory, 1991), or motion induced blindness (Bonneh et al., 2001). Other examples of the modulation of perception are illusions based on cognitive general rules, such as Ames window (Ittelson, 1952) and Hollow faces (Gregory, 1970; Hill and Bruce, 1993). In these illusions unusual objects or shapes give systematic errors, as they are in conflict with fixed rules or general knowledge.

Over the last few years, many studies claim to have proven CP, without the use of these illusions based on fixed rules or general knowledge. For example, studies show that a bottle of water looks closer when we are thirsty (Balcetis and Dunning, 2010), social expectations affect basic perceptual experiences, i.e., faces with African American features look darker (Levin and Banaji, 2006; Zhong and Leonardelli, 2008), and words are easier to detect when they are morally relevant (Gantman and Van Bavel, 2014). Most researchers consider these results as such pervasive evidence of cp, that the classical notion of cognitive impenetrability is often considered to be untenable.

Although many studies claim to have proven cp, Firestone and Scholl pointed out some significant problems in most of these experiments (Firestone and Scholl, 2015). They state that perceptual top-down research “falls prey to a set of pitfalls.” Roughly said, there are two major problems. The first problem within this field is that most results reflect top-down processes in early visual selection through attention shifts. Researches have shown that selection of input can be under top-down control, for instance through eye movements or attention shifts. However, it fails to prove that *after* selection the translation to percept is under top-down control. For example, inattentional blindness (Mack and Rock, 1998; Most et al., 2005; Ward and Scholl, 2015) might be a failure to see or memorize (Wolfe, 1999; Lamme, 2003) what we do not attend to. According to Lamme (2003), attention and conscious perception might be two separated systems, in which attention is needed to store our actual perception in working memory and to be able to report it afterward. According to this theory, it remains unclear whether inattentional blindness is a result of insufficient attention, insufficient perception or insufficient conscious memory.

The second problem is that experimental results are often not direct proof of change in perception *per se*, but are possibly a reflection of, for instance, our judgment. We can directly see that a bottle of water is closer when we are thirsty or just assume/conclude that it is closer. Another example is the study of Wesp and Gasper (2012). In an earlier experiment they found that less accurate throwing of darts led to estimation of smaller target-size, as if one’s performance perceptually resized the target (Wesp et al., 2004). However, when they replicated this experiment in 2012, subjects were told that the darts were defective. This additional instruction eliminated all correlation between performance and reported size of the target. This result indicates that if an experiment shifts perceptual reports, it could be possible that the shift reflects changes in judgment, rather than changes in perception. Other examples of studies that possibly do not reflect change in perception, although claiming to do so, are experiments using neuroimaging and electrophysiology. Although feedback connectivity in descending neural pathways are often interpreted as top-down effects, in which higher brain regions are assumed to modulate lower brain regions through descending neural pathways (Bar et al., 2006; Gilbert and Li, 2013), such imaging studies are per definition correlational. Specific neuronal interactions and feedback connectivity might be a reflection of our visual percept, but could also be a reflection of, for example, recall (Le Bihan et al., 1993) or imagery (Kosslyn, 2005). Thus, activation that is registered via an electrode or MRI scanner might be not always *necessary* or even not *directly* related to perception. Even when neuroimaging data do reflect a direct effect of feedback processing on perception, for example in unconscious inferences, this process is not under conscious control. Using neural data or behavioral data can be very useful in supporting perceptual changes by controlled top-down processes, however, it is not conclusive by itself.

The experimental pitfalls pointed out by Firestone and Scholl make it arguable whether perception is indeed cognitively penetrable or whether most of these studies are methodologically insufficient. The pitfalls listed by Firestone and Scholl mostly rest on the assumption that attention is pre-perceptual and memory is post-perceptual, and that it is often not clear which actual process has been affected. However, it is debatable whether attention and memory should be considered as purely pre- and post-perceptual systems, or as part of the mechanisms by which top-down processes influence the actual percept and thereby as part of the visual system (Lupyan, 2016).

We suggest that one can interpret cp in two different ways, in a broad and narrow sense. The broader sense of cp suggests that attention and memory are part of the visual system, and that top-down processes can influence the perceptual system. In this definition, perception is penetrable when top-down processes change attention, perception or memory. If cp is interpreted in the broad sense, many studies have provided fairly strong evidence of cp. For example, scene knowledge affects perception of edge orientations (Neri, 2014), knowledge of the real-world size of, e.g., a basketball affects apparent speed of motion (by altering perception of distance) (Andrés et al., 2015), knowledge of usual object colors shades our color perception (Hansen et al., 2006; Olkkonen et al., 2008; Witzel et al., 2011; Kimura et al., 2013) and influences the intensity of color afterimages (Lupyan, 2015a,b), and hearing the right word can make something visible that is otherwise invisible (Lupyan and Ward, 2013).

In the narrow sense of cp, however, the notion of cp is less obvious. We define narrow cp as follows. Narrow cp occurs when *flexible* factors (that can be learned and unlearned) affect perception, after the effects of attention, selection and memory are dismissed (see Vance and Stokes, 2016 for a similar definition). According to this narrow definition of cp, the pivotal question is whether selected malleable top-down factors can still affect perceptual experiences *after* sensory input (attention) and before reporting (memory). Two requirements need to be met before narrow cp is established. First, perception itself has to be unambiguously influenced by top-down processes. Second, these top-down processes must be flexible, in the sense that a healthy adult is able to turn these processes on and off, through training or voluntary decisions. Thus, fixed top-down processes (such as brightness perception being affected by surrounding information) do not count as examples of narrow cp.

In many of the previously mentioned studies (Hansen et al., 2006; Olkkonen et al., 2008; Witzel et al., 2011; Kimura et al., 2013; Neri, 2014; Martín et al., 2015; Witzel, 2016) purely attentional or post-perceptual processes (Lupyan and Ward, 2013) may have caused the observed effects. For instance, it could be argued that scene knowledge primarily affects orientation judgments, rather than that it causes perceptual distortions. Similarly, perhaps real world knowledge affects speed judgments more than that it creates actual illusions in speed perception. Furthermore, studies of binocular rivalry and continuous flash suppression have shown that attention/selection can determine the dominance of a stimulus (Chong et al., 2005). In other words, selection through attention may cause the effects of top-down processes on binocular rivalry and continuous flash suppression.

We acknowledge that it is very difficult to separate out attentional and perceptual effects. Some attention researchers may therefore not share our notion of narrow CP, since it could be argued that attention cannot be separated from perception. However, it is crucial to stress that in our definition of narrow CP, selection or amplifying effects of attention do not constitute narrow CP. These effects of attention on perception clearly occur and are consistently found in both behavior and neural activity. However, in our definition of narrow CP, flexible top-down factors should affect perception after selection has taken place, and in such a way that the contents of perception are altered (not merely the level of awareness). We assert that although it might be difficult, it is not impossible to prove narrow CP after the effects of attention, selection and memory are dismissed. For example, some illusions, such as the McGurk effect (McGurk and Macdonald, 1976), are clearly distortions of perception (from a first person perspective). In experiments without such a clear subjective distortion, it is hard to prove whether perception, or pre- or post-perceptual processes are affected. We, therefore, assert that in order to prove cp according to the narrow definition, we need to focus on perception from a first person perspective instead of (or in addition to) using proxies for perception. For example, by using clear visual illusions. Only when top-down, malleable, factors cause a clear illusion from a first person perspective, strong claims about the narrow definition of cp can be made.

Importantly, although visual illusions may be considered as proof for cp in the broader sense, awareness and understanding of the illusion cannot make them unseen and therefore most visual illusions cannot (yet) directly provide evidence for narrow cp. These illusions seem to be caused by fixed rules, which are hardwired into the visual system.

In conclusion, we claim that there is currently decisive evidence for CP when defined broadly, but not (yet) for CP in the narrower sense.

## Peripheral Illusions

Here we take a critical position toward the existence of narrow CP, i.e., the occurrence of *flexible*, learnable top-down factors affecting the contents of perception (as shown through clear illusions) while dismissing the effects of attention and memory. We want to point out, however, that the current lack of evidence for narrow cp does not necessarily imply that it does not exist. An alternative explanation for the absence of proof might be the fact that in nearly all illusion studies, stimuli are presented foveally, while they are attended. Since the signals from the fovea are often high fidelity, bottom-up input requires less or no direct top-down influences. In contrast to these clear foveal signals, the resolution in our peripheral vision is roughly equivalent to “looking through a frosted shower door” (Eagleman, 2001). We suggest that with noisy sensory input, like this peripheral frosted shower door, we have a much better chance of finding evidence that noisy bottom-up signals might be influenced by first-person factors, such as personal traits, experiences and believes. Even though sensory signals can also be ambiguous in the foveal part of the retina, as they confuse information from surfaces and illuminants, and because of the 3D to 2D projection, the essential difference between a noisy foveal image and an image in the periphery, is that in one case the external input is noisy, while in the other case the input is clear but the processing is noisy. The difference can be understood as follows. Imagine two reporters; one is a very reliable reporter while the other one is extremely chaotic and unreliable. When the very good reporter (i.e., the fovea) reports to the control room that the situation is disorderly, and there are riots everywhere, the control room will simply conclude that that is the current state of affairs. However, when the chaotic reporter (i.e., the peripheral signal) delivers an incoherent report, the control room will try to use best guesses to really understand what is going on. In other words, when the fovea reports to the brain that the external stimulus is noisy, the brain has no reason to override this report, and thus no flexible illusion will be created (only illusions based on fixed rules). However, when the fovea transmits a low fidelity report, the brain may augment this report, and thus possibly create a visual illusion based on transient cognition.

**FIGURE 1:**
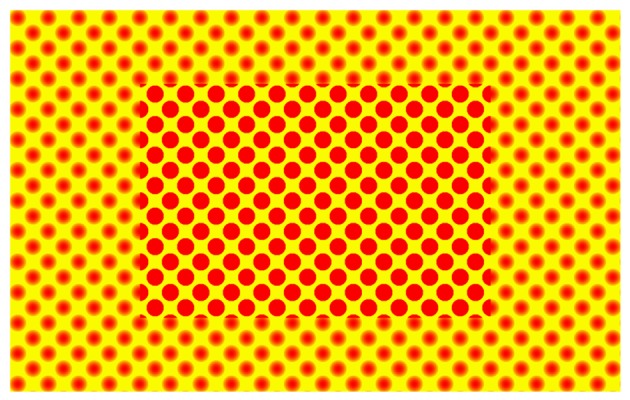
Uniformity illusion. (see also www.uniformillusion.com for more examples). The uniformity illusion becomes apparent when you fixate your eyes for a prolonged period of time on the center of the image. The peripheral dots take on the appearance of the central dots.

Peripheral vision becomes especially noisy during long fixations (Clarke, 1961; Martinez-Conde et al., 2006), in which (parts of) perception flexibly adopt a new identity based on global visual information and possibly high-level factors. Perhaps, peripheral illusions based on such long fixations could prove an effect of cognitive contents. They might be more sensitive to learnable priors and less driven by automatic algorithms, as their bottom-up signal is noisy. One striking visual illusion in peripheral vision is the uniformity illusion (see **Figure 1**)^1^. This illusion suggests that the detailed peripheral visual experience is partially based on a reconstruction of reality. In a visual display where central stimuli differ from peripheral stimuli on specific properties, central stimuli appear to overflow into the periphery for extended periods of time. Observers thus perceive the stimuli in the periphery to take on the properties of the central stimuli, resulting in a uniform field encompassing the center and the periphery of the display (Otten et al., 2016). This uniformity illusion has been demonstrated for a wide range of visual features, such as luminance, orientation, motion and texture. Importantly, unlike most other visual illusions, this is an illusion based on weak sensory processing. Although it seems likely that the illusion is (at least partly) driven by fixed rules and automatic algorithms just as other visual illusions are, more research is required in order to answer the question whether learnable priors can affect this illusion. Its ambiguous nature, its global effect on perception and the wide range of visual features in which this illusion occurs, provide the ideal circumstances to study how the brain constructs visual illusions and to what extent such illusions are cognitively penetrable.

To summarize, there are still some disagreements concerning the role of cognitive penetrability in visual perception. We do not debate the existence of CP in the broader sense. However, in our definition of narrow CP, attention and memory are considered to be pre-perceptual and post-perceptual processes. Moreover, cognitive penetrability only occurs when *flexible*, learnable factors affect the contents of perception, after the effects on attention and memory are dismissed. We argue that narrow cp has not (yet) been proved, since most evidence for cognitive penetration is based on methods that employ proxies for perception. The one data point that could really prove narrow cp is a clear illusion from a first-person perspective. However, so far, clear illusions do not support narrow cp, as these illusions cannot be unseen (i.e., they are driven by unchangeable rules).

To provide more insight into the matter, future research should focus on cognitively induced perceptual illusions when the sensory signal is noisy, such as during the uniformity illusion. Interesting research questions would be; which functional manipulations affect the uniformity illusion? Or, how can prior expectations influence this illusion? For example, when subjects are divided into two categories, in which subjects of category one are given no priors and subjects of category two are first given correct priors about the stimuli in the periphery, followed by false priors. Can these correct/false priors strengthen/weaken the uniformity illusion? And does changing the priors within subjects change the perception of the same stimuli? If future research indeed verifies that illusions can be affected through learnable cognitive priors when sensory input is unreliable, then the notion of cognitive penetrability receives clear proof, even when it is defined narrowly. However, if even under these circumstances narrow cp does not occur, then it becomes doubtful whether narrow CP exists at all. In that case, the effects of cognition are probably either purely based on post-perceptual processes (e.g., memory, judgment) or pre-perceptual processes (input selection), or driven by fixed, unlearnable factors.

## Author Contributions

All authors shared the opinion of a critical position toward cognitive penetrability in visual perception, and the significance of cognitively induced perceptual illusions in peripheral vision. NL drafted the manuscript, which was adjusted based on feedback from YP and EdH. All authors approve the manuscript.

## Conflict of Interest Statement

The authors declare that the research was conducted in the absence of any commercial or financial relationships that could be construed as a potential conflict of interest.

## Footnotes

1 http://www.uniformillusion.com

## References

[B1] Adelson E. H. (1993). Perceptual organization and the judgment of brightness. *Science* 262 2042–2044. 10.1126/science.8266102 8266102

[B2] Anderson S. J. Mullent K. T. Hesst R. F. Anderson S. J. Mullen K. T. Hess R. F. (1991). Human peripheral spatial resolution for achromatic and chromatic stimuli: limits imposed by optical and retinal factors. *J. Physiol.* 442 47–64. 10.1113/jphysiol.1991.sp018781 1798037 PMC1179877

[B3] Andrés M. Chambeaud J. G. Barraza J. F. (2015). The effect of object familiarity on the perception of size and distance. *J. Exp. Psychol. Hum. Percept. Perform.* 21 239–247. 10.1037/xhp0000027 25665088

[B4] Balcetis E. Dunning D. (2010). Wishful seeing: more desired objects are seen as closer. *Psychol. Sci.* 21 147–152. 10.1177/0956797609356283 20424036

[B5] Bar M. (2004). Visual objects in context. *Nat. Rev. Neurosci.* 5 617–629. 10.1038/nrn1476 15263892

[B6] Bar M. Kassam K. S. Ghuman A. S. Boshyan J. Schmid A. M. Dale A. M. (2006). Top-down facilitation of visual recognition. *Proc. Natl. Acad. Sci. U.S.A.* 103 449–454. 10.1073/pnas.0507062103 16407167 PMC1326160

[B7] Block N. (2007). Consciousness, accessibility, and the mesh between psychology and neuroscience. *Behav. Brain Sci.* 30 481–548. 10.1017/S0140525X07002786 18366828

[B8] Block N. (2011). Perceptual consciousness overflows cognitive access. *Trends Cogn. Sci.* 15 567–575. 10.1016/j.tics.2011.11.001 22078929

[B9] Bonneh Y. S. Cooperman A. Sagi D. (2001). Motion-induced blindness in normal observers. *Nature* 411 798–801. 10.1038/35081073 11459058

[B10] Chong S. C. Tadin D. Blake R. (2005). Endogenous attention prolongs dominance durations in binocular rivalry. *J. Vis.* 5 1004–1012. 10.1167/5.11.6 16441198

[B11] Clark A. (2013). Whatever next? Predictive brains, situated agents, and the future of cognitive science. *Behav. Brain Sci.* 36 181–204. 10.1017/S0140525X12000477 23663408

[B12] Clarke F. J. J. (1961). Visual recovery following local adaptation of the peripheral retina (Troxler’s Effect). *Opt. Acta Int. J. Opt.* 8 121–135. 10.1080/713826370

[B13] Eagleman D. M. (2001). Visual illusions and neurobiology. *Nat. Rev. Neurosci.* 2 920–926. 10.1038/35104092 11733799

[B14] Firestone C. Scholl B. J. (2015). Cognition does not affect perception: evaluating the evidence for “top-down” effects. *Behav. Brain Sci.* 39:e229 10.1017/S0140525X15000965 26189677

[B15] Fodor J. A. (1983). *The Modularity of Mind an Essay on Faculty Psychology.* Cambridge, MA: MIT Press 10.2307/2184717

[B16] Gantman A. P. Van Bavel J. J. (2014). The moral pop-out effect: enhanced perceptual awareness of morally relevant stimuli. *Cognition* 132 22–29. 10.1016/j.cognition.2014.02.007 24747444

[B17] Gibson J. J. (1966). *The Senses Considered as Perceptual Systems.* Boston, MA: Houghton Mifflin 266–287.

[B18] Gilbert C. D. Li W. (2013). Top-down influences on visual processing. *Nat. Rev. Neurosci.* 14 350–363. 10.1038/nrn3476 23595013 PMC3864796

[B19] Gregory R. L. (1970). *The Intelligent Eye.* London: Weidenfeld & Nicolson.

[B20] Hansen T. Olkkonen M. Walter S. Gegenfurtner K. R. (2006). Memory modulates color appearance. *Nat. Neurosci.* 9 1367–1368. 10.1038/nn1794 17041591

[B21] Hill H. Bruce V. (1993). Independent effects of lighting, orientation, and stereopsis on the hollow-face illusion. *Perception* 22 887–897. 10.1068/p220887 8190593

[B22] Ittelson W. H. (1952). *The Ames Demonstrations in Perception: A Guide to their Construction and Use.* New Jersey, NJ: Princeton University Press.

[B23] Kimura A. Wada Y. Masuda T. Goto S. Tsuzuki D. Hibino H. (2013). Memory color effect induced by familiarity of brand logos. *PLoS ONE* 8:e68474 10.1371/journal.pone.0068474 PMC3707833 23874638

[B24] Knill D. C. Pouget A. (2004). The Bayesian brain: the role of uncertainty in neural coding and computation. *Trends Neurosci.* 27 712–719. 10.1016/j.tins.2004.10.007 15541511

[B25] Kosslyn S. (2005). Mental images and the brain. *Cogn. Neuropsychol.* 22 333–347. 21038254 10.1080/02643290442000130

[B26] Lamme V. A. F. (2003). Why visual attention and awareness are different. *Trends Cogn. Sci.* 7 12–18. 10.1016/S1364-6613(02)00013-X 12517353

[B27] Lamme V. A. F. (2006). Towards a true neural stance on consciousness. *Trends Cogn. Sci.* 10 494–501. 10.1016/j.tics.2006.09.001 16997611

[B28] Le Bihan D. Turner R. Zeffiro T. A. Cuénod C. A. Jezzard P. Bonnerot V. (1993). Activation of human primary visual cortex during visual recall: a magnetic resonance imaging study. *Proc. Natl. Acad. Sci. U.S.A.* 90 11802–11805. 8265629 10.1073/pnas.90.24.11802 PMC48072

[B29] Levin D. T. Banaji M. R. (2006). Distortions in the perceived lightness of faces: the role of race categories. *J. Exp. Psychol. Gen.* 135 501–512. 10.1037/0096-3445.135.4.501 17087569

[B30] Lupyan G. (2015a). Cognitive penetrability of perception in the age of prediction: predictive systems are penetrable systems. *Rev. Philos. Psychol.* 6 547–569. 10.1007/s13164-015-0253-4

[B31] Lupyan G. (2015b). Object knowledge changes visual appearance: semantic effects on color afterimages. *Acta Psychol.* 161 117–130. 10.1016/j.actpsy.2015.08.006 26386775

[B32] Lupyan G. (2016). Not even wrong: the “it’s just X” fallacy. *Behav. Brain Sci.* 39:e251 10.1017/S0140525X15002721 28355864

[B33] Lupyan G. Ward E. J. (2013). Language can boost otherwise unseen objects into visual awareness. *Proc. Natl. Acad. Sci. U.S.A.* 110 14196–14201. 10.1073/pnas.1303312110 23940323 PMC3761589

[B34] Mack A. Rock I. (1998). *Inattentional Blindness.* Cambridge, MA: MIT Press.

[B35] Martinez-Conde S. Macknik S. L. Troncoso X. G. Dyar T. A. (2006). Microsaccades counteract visual fading during fixation. *Neuron* 49 297–305. 10.1016/j.neuron.2005.11.033 16423702

[B36] Martín A. Chambeaud J. G. Barraza J. F. (2015). The effect of object familiarity on the perception of motion. *J. Exp. Psychol. Hum. Percept. Perform.* 41 283–288. 10.1037/xhp0000027 25665088

[B37] McGurk H. Macdonald J. (1976). Hearing lips and seeing voices. *Nature* 264 691–811. 10.1038/264746a0 1012311

[B38] Most S. B. Scholl B. J. Clifford E. R. Simons D. J. (2005). What you see is what you set: sustained inattentional blindness and the capture of awareness. *Psychol. Rev.* 112 217–242. 10.1037/0033-295X.112.1.217 15631594

[B39] Neri P. (2014). Semantic control of feature extraction from natural scenes. *J. Neurosci.* 34 2374–2388. 10.1523/JNEUROSCI.1755-13.2014 24501376 PMC3913878

[B40] Olkkonen M. Hansen T. Gegenfurtner K. R. (2008). Color appearance of familiar objects: effects of object shape, texture, and illumination changes. *J. Vis.* 8 13.1–13.16. 10.1167/8.5.13 18842084

[B41] Otten M. Pinto Y. Paffen C. L. E. Seth A. K. Kanai R. (2016). The uniformity illusion: central stimuli can determine peripheral perception. *Psychol. Sci.* 28 56–68. 10.1177/0956797616672270 28078975

[B42] Pinto Y. van Gaal S. de Lange F. P. Lamme V. A. Seth A. K. (2015). Expectations accelerate entry of visual stimuli into awareness. *J. Vis.* 15:13 10.1167/15.8.13. 26114676

[B43] Rahnev D. Maniscalco B. Graves T. Huang E. de Lange F. P. Lau H. (2011). Attention induces conservative subjective biases in visual perception. *Nat. Neurosci.* 14 1513–1515. 10.1038/nn.2948 22019729

[B44] Ramachandran V. S. Gregory R. L. (1991). Perceptual filling in of artificially induced scotomas in human vision. *Nature* 350 699–702. 10.1038/350699a0 2023631

[B45] Summerfield C. de Lange F. P. (2014). Expectation in perceptual decision making: neural and computational mechanisms. *Nat. Rev. Neurosci.* 15 745–756. 10.1038/nrn3838 25315388

[B46] Vance J. Stokes D. (2016). Noise, uncertainty, and interest: predictive coding and cognitive penetration. *Conscious. Cogn.* 47 86–98. 10.1016/j.concog.2016.06.007 27329550

[B47] Ward E. J. Scholl B. J. (2015). Inattentional blindness reflects limitations on perception, not memory: evidence from repeated failures of awareness. *Psychon. Bull. Rev.* 22 722–727. 10.3758/s13423-014-0745-8 25515671

[B48] Wesp R. Cichello P. Gracia E. B. Davis K. (2004). Observing and engaging in purposeful actions with objects influences estimates of their size. *Percept. Psychophys.* 66 1261–1267. 10.3758/BF03194996 15813192

[B49] Wesp R. Gasper J. (2012). Is size misperception of targets simply justification for poor performance? *Perception* 41 994–996. 10.1068/p7281 23362677

[B50] Westheimer G. (1982). The spatial grain of the perifoveal visual field. *Vision Res.* 22 157–162. 10.1016/0042-6989(82)90177-8 7101739

[B51] Witzel C. (2016). An easy way to show memory colour effects. *IPerception* 7 1–11. 10.1177/2041669516663751 PMC5030741 27698988

[B52] Witzel C. Valkova H. Hansen T. Gegenfurtner K. R. (2011). Object knowledge modulates colour appearance. *Iperception* 2 13–49. 10.1068/i0396 23145224 PMC3485772

[B53] Wolfe J. M. (1999). “Inattentional amnesia,” in *Fleeting Memories* ed. Coltheart V. (Cambridge, MA: MIT Press) 71–94. 10.1162/0898929053747685

[B54] Zhong C. B. Leonardelli G. J. (2008). Cold and lonely: does social exclusion literally feel cold? *Psychol. Sci.* 19 838–842. 10.1111/j.1467-9280.2008.02165.x 18947346

